# Dietary Supplement Use in Relation to Socio-Demographic and Lifestyle Factors, including Adherence to Mediterranean-Style Diet in University Students

**DOI:** 10.3390/nu14132745

**Published:** 2022-06-30

**Authors:** Ewa Sicinska, Dawid Madej, Maria Karolina Szmidt, Olga Januszko, Joanna Kaluza

**Affiliations:** Department of Human Nutrition, Institute of Human Nutrition Sciences, Warsaw University of Life Sciences–SGGW, 02-787 Warsaw, Poland; dawid_madej@sggw.edu.pl (D.M.); maria_szmidt@sggw.edu.pl (M.K.S.); olga_januszko@sggw.edu.pl (O.J.); joanna_kaluza@sggw.edu.pl (J.K.)

**Keywords:** determinants, dietary supplements, Mediterranean diet score, university students

## Abstract

The study aimed to examine socio-demographic and lifestyle determinants, including diet quality, of dietary supplement (DS) use among 2545 students who attended public universities in Warsaw. The data was collected using a self-administered health and lifestyle questionnaire and a 5-day dietary record method. Diet quality was assessed using a Mediterranean Diet Score. To determine the covariates of DS use, multivariate-adjusted logistic regression models with an estimation of odds ratios (ORs) and 95% confidence intervals (95% CIs) were used. The results showed that 41% of participants were DS users. The following predictors of DS use were identified: gender (male vs. female; OR:0.62, 95% CI:0.50–0.79), physical activity (high vs. low; OR:1.79, 95% CI:1.45–2.20), BMI (≥25 vs. 18.5–24.9 kg/m^2^; OR:0.77, 95% CI:0.61–0.98), cigarette smoking (yes vs. no; OR:0.67, 95% CI:0.52–0.86), and presence of chronic diseases (yes vs. no; OR:2.37, 95% CI:1.89–2.98). Moreover, higher nutritional knowledge, special diet usage, eating more meals/day, and fortified food consumption were determinants of DS use. Adherence to the Mediterranean-style diet was positively associated with DS use, a 1-score increment was associated with a 10% (*p*-trend = 0.011) higher probability of DS usage. Given that the use of vitamins and/or minerals is common among university students and their users are characterized by eating a higher quality diet, investigating the contribution of DS in overall dietary nutrient intake warrants further study.

## 1. Introduction

The use of dietary supplements (DS) is widespread in developed countries. In the USA, the use of these products is declared by 58% of the adult population [1], and in Europe from 2% to 66% depending on the country and gender [2]. Vitamins and/or minerals are the most commonly used supplements [1,2]. Overall, DS users differ from non-users in socio-demographic characteristics and lifestyle. Supplement use has been reported to increase with age, income, education, and women are more likely to use supplements than men [3]. In addition, people using DS are characterized by health-related habits, including better dietary patterns, higher physical activity, maintenance of normal body weight, and tobacco avoidance [3]. However, most of the studies on factors of DS usage are based on broad age groups; despite that, the reasons for their use vary depending on age [3]. For example, while the use of DS by school children reflects their parents’ views [4], university students use these products of their own free will, depending on nutritional knowledge and lifestyle [5]. Some university students do not eat meals regularly due to organizational difficulties, such as extracurricular activities or part-time jobs. It makes them feel that they are not getting enough vitamins and minerals in their diet, which might be one reason for them to use DS to boost their health [5]. However, the health benefits and risks of using DS are controversial. DS with nutrients can benefit some at-risk groups when meeting nutrient needs from the diet alone is difficult due to increased requirements, dietary restrictions, or intolerances. On the other hand, excessive intakes of some DS are associated with an increased morbidity and mortality risk in the population [6]. Therefore, understanding the connection between DS usage and diet quality is essential to determine the effectiveness of supplement use in a variety of populations, including university students.

Most studies conducted among university students focused on the reasons and types of supplements used [5,7,8,9], but less frequently on the relationship with socio-demographic and lifestyle factors. Furthermore, there is little research using a holistic approach for the estimation of diet quality among those who use these products [10] and an existing study in this area was limited mainly to broader age groups or older adults [11,12,13,14]. Therefore, the study aimed to examine socio-demographic and lifestyle determinants including diet quality on DS use among university students. Using the Mediterranean Diet Score as a diet quality indicator, we examined whether better diet quality is associated with a more common use of DS.

## 2. Materials and Methods

### 2.1. Study Design

The cross-sectional study was carried out from October 2013 to April 2020 among students attending public universities in Warsaw. Volunteers were recruited through advertisements at the campus of universities as well as on social media. The inclusion criteria for participating in the study were: student status in a public university and the age of respondents from 18 to 35 years old. The exclusion criteria consisted of pregnancy or lactation, disease occurrence that requires special dietary treatment, and incomplete or incorrectly filled out questionnaires.

All study procedures were carried out in line with the Helsinki Declaration (1964) and its later amendments. The Ethical Committee of the Warsaw University of Life Sciences approved the study in 2016 (Resolution No. 09_1/2016). According to Polish law, such approval in non-invasive studies was not required before this year.

### 2.2. Study Population and Data Collection

**Figure 1:**
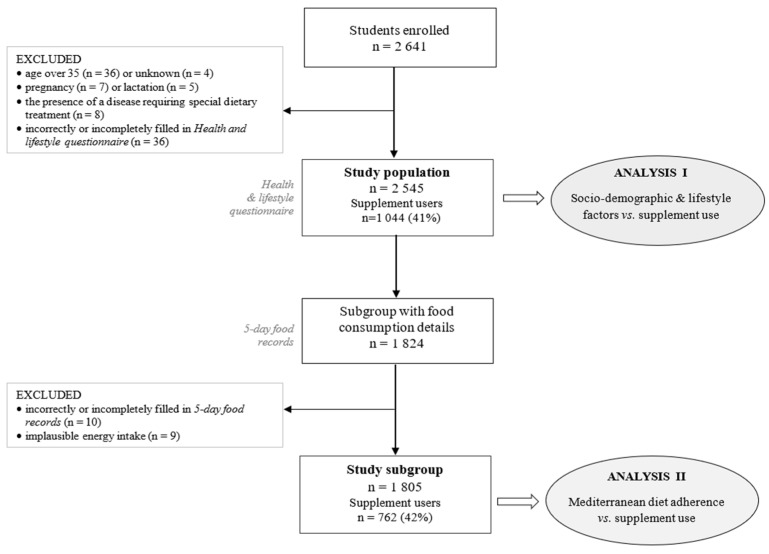
Study population flow chart.

The data was collected using two questionnaires: a self-administered health and lifestyle questionnaire in the entire study group, and a 5 day dietary record method in a subgroup (data collected since 2016). The most significant number of respondents (81.8%) attended four universities: Warsaw University of Life Sciences (WULS-SGGW), the University of Warsaw (UW), Warsaw University of Technology (WUT), and the Medical University of Warsaw (MUW). A total of 2641 students volunteered for the study; however, 60 participants were found not to meet the inclusion criteria, and 36 were excluded due to incorrectly or incompletely filling out the health and lifestyle questionnaire (i.e., at least one whole section was missing in the questionnaire). Ultimately, 2545 respondents were included in the study (Figure 1, Analysis I).

### 2.3. Health and Lifestyle Assessment

The health and lifestyle questionnaire included 31 questions and consisted of the following sections: (1) socio-demographic characteristics (2) health and lifestyle status (3) self-reported level of nutritional knowledge and eating habits (4) DS usage, as well as (5) voluntarily fortified foods usage. The questionnaire was developed at the Department of Human Nutrition (WULS-SGGW, Poland) for an earlier study of adults, verified among students, and was adapted for different age groups [4,15]. The Health and Lifestyle Questionnaire is presented in Supplementary Material.

Socio-demographic questions regarded data on age, gender, place of living and socioeconomic status. Health and lifestyle questions provided data on self-reported health status, cigarette smoking, occurrence of chronic diseases, self-reported physical activity levels, as well as weight and height. Respondents assessed their physical activity level by choosing from one to five predefined categories, ranging from a very low to a very high level. To help respondents choose the appropriate category of physical activity, each category gave examples of types of exercise and the number of hours spent weekly on these activities. The body mass index was calculated by dividing self-reported weight by height squared and interpreted according to the World Health Organization classification [16].

Respondents were given a choice of seven predefined opinions about their nutritional knowledge, ranging from “no knowledge at all” to “excellent.” Questions on eating habits included whether students followed a special diet, the type of diet, the period over which it was followed, and the number of meals consumed during the day. The question on voluntarily fortified product usage included a definition of these products and gave some examples of them.

### 2.4. Assessment of the DS Use

Participants were asked about all DS taken on the test day and during the 6 months before the study. Information was collected on the name and brand, the form used (i.e., capsules, tablets, powder, etc.), duration of use and the reason for usage. A DS user was considered a person who used at least one supplement for at least one week or longer over 6 months. DS were categorized into 7 subgroups: (1) single vitamin (2) single mineral (3) multivitamin/mineral(s) (4) multi-ingredient DS containing vitamin/mineral(s) plus herb extracts (5) polyunsaturated fatty acids (PUFA) (6) proteins or protein-carbohydrate (7) others. The duration of DS use was defined as short-term (from 7 days to less than 1 month), medium-term (1–3 months), and long-term (longer than 3 months). Respondents who declared DS use were asked to mark one or more predefined reasons for their usage or could choose to list their own reason. When the respondents declared not using DS, they were also asked to indicate one or more of the predefined reasons for not taking them, as well as being able to list their own reason.

### 2.5. Food Consumption Assessment

The 5-day food record method was used to collect data about food consumption, covering four non-consecutive weekdays and one weekend day (Figure 1, Analysis II). To encourage participation in the food consumption records, free nutritional advice was offered to those who completed them. The respondents were instructed on how to provide detailed information about the food and drinks consumed and determine the portion sizes (using kitchen scales or household measures). Participants were asked to return their food record questionnaires within 3 weeks of the recruitment meeting. Qualified nutritionists or dieticians verified the returned questionnaires. Imprecise or missing information on portion size were corrected together with the respondent using a photo album of products and dishes. Of the 1824 participants who returned the completed food record questionnaires, we excluded those who filled them in incorrectly or incompletely (recorded less than 3 days; n = 10), and with implausible values for total energy intake (±3 standard deviations from the mean value of the log-transformed energy, calculated separately for men and women; n = 9). The data from the food record questionnaires were entered into the “DIETA 6” program (Polish National Institute of Public Health-PZH). To estimate the content of energy and nutrients in respondents’ diets, the software uses Polish tables of composition and nutritional value [17].

### 2.6. Adherence to Mediterranean-Style Diet

Diet quality was assessed by calculating the Mediterranean Diet Score (MDS). For each presumably beneficial diet component (vegetables, legumes/seeds/nuts, fruits, cereals, fish, and seafood, the ratio of monosaturated to saturated fatty acids, a value of 0 was assigned for consumption below and a value of 1 for intake at or above the sex-specific median (Table S1). For foods considered unfavourable in excess (dairy products, meat and meat products), a value of 0 was assigned for consumption at or above, and 1 for consumption below the sex-specific median. For ethanol consumption, a value of 1 was assigned for intake of 5–25 g/day for women and 10–50 g/day for men, and a value of 0 otherwise. The total MDS consisted of a sum of the scores for each component and ranged from 0 (a lack of adherence) to 9 points (a high adherence to the Mediterranean-style diet) [18].

### 2.7. Statistical Analysis

To state the differences in baseline characteristics between DS users and non-users, the Student t-test was performed for continuous variables with normal distribution and the Chi-square test was used for categorical variables. Based on the Kolmogorov-Smirnov test, the hypothesis of normality of nutritional variables (daily energy, nutrients, and food consumption) was rejected; therefore, the Mann-Whitney U test was used to determine statistically significant differences between DS users and non-users.

To explore the associations between DS usage and possible predictors of their use, two logistic regression models (odds ratios, ORs with 95% confidence intervals, 95% CIs) were calculated: the univariate (Model 1: crude data) and multivariate-adjusted (Model 2). In addition, according to the backward elimination method, the stepwise regression model (Model 3) was used to select only statistically significant variables associated with DS usage. DS non-users constituted a referent category in logistic regression models. The Hosmer-Lemeshow test was used to assess the models’ goodness-of-fit.

Multivariate-adjusted models included potential determinants of DS usage, i.e., the age of participants (continuous variable), gender (female or male), place of living (village, small town, or city), self-reported socioeconomic status (very good/good, or average/poor), self-reported physical activity level (low, moderate, or high), body mass index (<18.5, 18.5–24.9, or ≥25 kg/m2), self-reported health status (at least good, or average/poor), smoking cigarettes (no, yes), presence of chronic diseases (no, yes), self-reported nutritional knowledge level (low, average, or high), using special diet (no, yes), the number of meals consumed per day (≤3, 4, or ≥5), fortified food consumption (no, yes). Missing data and answering “I don’t know” for the number of meals per day (32.9%), nutritional knowledge level (2.0%) and fortified food consumption (20.6%) were modelled as separate categories.

Moreover, multivariate logistic regression was used to identify the association between DS usage and MDS as well as the score components. The score components and MDS were divided into four groups using quartile distribution as cut-offs, where the first quartile constituted a reference group. The model was adjusted for those variables that were statistically significant in Model 3 (the stepwise regression model).

All statistical analyses were performed using the SPSS version 27.0 (IBM, New York, NY, USA). *p*-values ≤ 0.05 were admitted as statistically significant.

## 3. Results

### 3.1. Characteristics of the Study Population

Over the past 6 months before the study, 41.0% of participants used DS, while 21.1% used these products on the test day. The majority of respondents were classified as medium-term DS users (1–3 months) (54.3%), followed by long-term users (longer than 3 months) (34.8%) and lastly short-term users (from 7 days to less than 1 month) (10.9%). Socio-demographic and lifestyle parameters stratified by DS usage in the university students’ population are presented in Table 1. Compared to non-users, there were statistically significantly more women among those who used DS (73.9% vs. 82.2%, respectively). DS users vs. non-users more frequently evaluated their physical activity as moderate or high (64.1% vs. 56.5% respectively), health status as average or poor (14.2% vs. 11.5%, respectively), and suffered from chronic diseases (23.7% vs. 11.1%, respectively), while less frequently declared smoking cigarettes (11.6% vs. 18.5%, respectively). It was found that significantly more users vs. non-users used a special diet (26.7% vs. 13.1%, respectively), evaluated their nutritional knowledge as high (51.8% vs. 38.5%, respectively), and ate more than 3 meals during the day (55.8% vs. 44.3%, respectively). Moreover, the consumption of fortified foods was more frequently declared by DS users vs. non-users (54.7% vs. 45.0%, respectively).

### 3.2. Types and Period of DS Use and Reason for the Use or Non-Use

The most frequently used type of DS were single vitamins such as vitamin D (25.3% of DS-users) and vitamin C (9.7%), whereas multi-ingredient supplements containing vitamins and/or minerals with or without herbs were used less commonly (Table 2). Usage of more than one DS during the past 6 months declared 45.7% of DS users. As the main reasons for DS use, respondents indicated “improve overall health” and “diet poor in nutrients”, while DS non-users pointed out that there is “no need to use it because of proper nutrition” and “a lack of effect on health improvement”.

### 3.3. Socio-Demographic and Lifestyle Determinants (Analysis I)

Multivariate-adjusted odds ratios (ORs) of DS use by socio-demographic and lifestyle factors in university students are presented in Table 3. DS users vs. non-users were less likely to be male (OR: 0.62, 95% CI: 0.50–0.79). Compared to respondents with a low physical activity level, those with high physical activity had a significantly higher ratio of odds for DS use (OR: 1.79, 95% CI: 1.45–2.20). Supplements were used less likely by overweight or obese students (BMI ≥ 25 kg/m^2^; OR: 0.77, 95% CI: 0.61–0.98) compared to those with a normal BMI (18.5–24.9 kg/m^2^). An inverse statistically significant trend between BMI and supplement usage was observed; each 1 kg/m^2^ increment in BMI was associated with a 4% (95% CI: 1–7%; *p*-trend = 0.003) lower probability of DS use. Supplements were used less likely by cigarette smokers than by non-smokers (OR: 0.67, 95% CI: 0.52–0.85). Students who suffer from chronic diseases were more likely to be supplement users (OR: 2.37, 95% CI: 1.89–2.98) than those without chronic diseases. In comparison to respondents with a low nutritional knowledge level, those with average as well as high nutritional knowledge had significantly higher ratios of odds for DS use (OR: 1.49, 95% CI: 1.09–2.04 and OR: 2.09, 95% CI: 1.51–2.88, respectively). Respondents who used a special diet had a 2-fold (OR: 2.00 95% CI: 1.61–2.49) higher probability of usage of DS compared to those who did not declare the use of a special diet. Students who consumed 4 meals per day were 1.3-fold (OR: 1.32, 95% CI: 1.09–1.61) more likely to be supplement users compared to students who ate ≤3 meals per day. Students who consumed fortified food had a 1.6-fold (OR: 1.64, 95% CI: 1.35–2.00) higher probability of usage of DS compared to those who were fortified food non-consumers.

### 3.4. Food Consumption and Mediterranean-Style Diet Adherence (Analysis II)

Daily energy, nutrients, and food consumption among university students by using DS were presented in Table 4. Compared with non-users, DS users consumed a smaller proportion of energy from carbohydrates, but a greater proportion from protein. DS users had higher daily consumption than non-users of vegetables (312 vs. 256 g, respectively), legumes, seeds and nuts (25.2 vs. 14.5 g, respectively), and fruits (253 vs. 232 g, respectively), but a lower consumption of cereals (180 vs. 191 g, respectively), meat and meat products (112 vs. 119 g, respectively). Compared with non-users, DS users’ diet was characterized by a higher monosaturated to saturated fatty acids ratio (1.19 vs. 1.24, respectively), while no significant differences between the groups were observed for the consumption of fish and seafood, dairy products, and ethanol intake. Adherence to a Mediterranean-style diet differed statistically significantly between supplement users and non-users; DS users were characterized by a higher value of MDS than non-users (5.16 vs. 4.78 points, respectively).

The likelihood of using DS across quartiles of Mediterranean-style diet components was presented in Table 5. The respondents in the highest quartile of vegetable consumption were more likely to be supplement users (OR: 1.76, 95% CI: 1.29–2.42; *p*-trend = 0.002) than those in the lowest quartile. In comparison to respondents in the first quartile of legumes, seeds and nuts consumption, those in the second, third, and fourth quartiles had significantly higher ratios of odds for DS usage (OR: 1.45, 95% CI: 1.07–1.96; OR: 1.35, 95% CI: 1.01–1.82; and OR: 2.01, 95% CI: 1.47–2.76, respectively; *p*-trend = 0.012). For fruit, the relationship was reversed; the respondents in the highest quartile compared to those in the lowest quartile of fruit consumption were less likely to be supplement users (OR: 0.67, 95% CI: 0.49–0.92; *p*-trend = 0.005). No significant differences between the extreme quartiles were found for cereals, fish and seafood, dairy products, meat and meat product consumption, as well as for the monosaturated to saturated fatty acids ratio and ethanol intake.

A statistically significant association between DS use and adherence to the Mediterranean-style diet was observed (Table 5). More respondents in the highest compared to the lowest quartile of MDS (6–9 vs. 1–3 points) were DS users-49.8% vs. 32.7%, respectively. The multivariate-adjusted OR between the extreme quartiles of MDS was 1.42, (95% CI: 1.04–1.93). A statistically significant trend between MDS and supplement usage was observed; each 1-point increment in the score was associated with a 10% (95% CI: 2–19%; *p*-trend = 0.011) higher probability of DS usage.

## 4. Discussion

The study showed that the use of supplements is common in university students and many socio-demographic and lifestyle determinants are associated with their use. Participants who used DS compared to non-users were less likely to be men, had a higher physical activity level, a lower risk of being overweight or obese, were less likely to be smokers and suffered from chronic diseases more frequently. In addition, higher nutritional knowledge, the usage of a special diet, eating more meals per day, the consumption of fortified food, and following a Mediterranean-style diet were determinants of DS use.

Prevalence of DS use was over 40% in the study population, and the most popular supplements consumed were single vitamins, followed by multivitamins and/or minerals. In studies conducted among Japanese [5], New Zealand [19], Italian [8], Croatian [7], and US [10] university students, the prevalence of DS use ranged from 17% to 66%. Similarly to our research, in the majority of those studies, the most commonly used DS by students were vitamins and minerals [5,8,9,10]. In some studies, protein/amino acids, fish oil/omega-3 supplements and weight loss supplements were popular as well [5,7,9].

In this study, the main motivation for using DS indicated by most of the students was “improving overall health” as well as enriching a “diet poor in nutrients”. Likewise, studies in Croatian [7], North American [9] and Japanese [5] university students found that the most commonly reported reasons were improving or promoting health. In addition, in those studies, other different reasons were pointed out, i.e., “the treatment of a specific disease or health problem”, “a recommendation by family or friends”, “an increase in energy”, “losing weight”, “building muscle”, and “enhancing performance” [5,7,9], which could be related to specific supplements used by respondents, nutritional knowledge or advertising these products.

Concerning the socio-demographic and lifestyle profile of DS users, the results of several studies conducted in developed countries among adult populations indicated that supplement users versus non-users were more likely to be women [11,13,20,21], older [11,13,21], living in a larger town/urban area [20,21], with a higher education level [13,20], reporting a chronic condition more frequently [13,21], and less likely to be smokers [12,13,21]. A lower BMI [8,13,21], a higher physical activity level [8,11,12,13,20], and using a special diet [11,13] among users than non-users was also reported. The majority of DS use predictors found in our study are consistent with the above-mentioned study results, excluding age, the place of residence, and educational level; however, our study was conducted in a relatively narrow age group (18–35 years old), among respondents who were in the process of getting a university education. The positive association of nutritional knowledge and DS use found in our study was consistent with findings obtained in a large sample of the French adult population, in which, compared with non-users, DS users were more likely to have greater knowledge of nutritional recommendations [13].

In this study, health status was not a predictor of DS use. The findings of other studies in this area are inconsistent, which may result from differences in the study populations, the definitions of DS users and health status assessment methods. Data from the European Prospective Investigation into Cancer and Nutrition (EPIC) study showed that DS consumption was higher in those who perceived their health as moderate or poor compared to those reporting excellent or good health [2]. On the other hand, in the large US study (National Health Interview Survey), vitamin/mineral supplement users reported better overall health than non-users despite apparent differences in clinically measurable health outcomes. Thus, it seems that the widespread use of DS in adults may be a result of an individual’s positive expectation that such product use leads to better health outcomes [22].

Taking into consideration that many of the analyzed factors were associated with DS use, it was surprising that no significant association between socioeconomic status and DS use was found. Such associations were observed in other studies, for example in the Canadian Community Health Survey (CCHS)-Nutrition [21] or in the National Health and Nutrition Examination Survey (NHANES) survey [1]. However, the current study was based on a self-assessment of socioeconomic status, and exact family income was not measured–this methodological issue could have had an impact on the findings.

In the study population, DS users, compared to non-users, showed a higher consumption of health-promoting products: vegetables, fruits, legumes, seeds, and nuts, and a lower consumption of meat and meat products. Moreover, adherence to the Mediterranean-style diet was positively associated with DS use; a 1-score increment in MDS was associated with a 10% higher probability of DS usage. Our findings are consistent with the results of other studies conducted among adults, where supplement use was associated with more healthy food patterns based on a higher consumption of vegetables, fruits, whole grains, pulses, fish, seafood and lower intakes of unprocessed and processed meat, offal, cakes, and alcoholic beverages [13] as well as based on higher diet quality scores, i.e., MDS [12,14], the Healthy Eating Index [11], and the Dietary Guideline Index [10]. In the study, MDS was selected for dietary pattern analysis due to its common use, being recognised by researchers, and its significant positive associations with healthy indicators in many studies [23]. Our results confirm the paradox called the ‘inverse supplement hypothesis’, whereby subjects most likely to use DS were those who need them least [24].

The present study has several strengths, including a large number of participants as well as a large number of DS users and detailed characteristics of respondents by socio-demographic and lifestyle factors. Additionally, detailed data on food consumption were obtained from the large subgroup of respondents using the 5-day food record method. The returned questionnaires were accurately checked by qualified nutritionists or dieticians and possible missing or imprecise information about the food consumed was completed. It allowed us to use logistic regression models to determine factors significantly associated with DS, also including the aspect of a Mediterranean-style diet adherence in the analysis.

The limitation of the study was its long period of investigation (7 years), during which the emergence of new DS on the market could have impacted their consumption. Moreover, 3% of students’ food records were returned after a lockdown was introduced due to the COVID-19 pandemic; this could also have affected the results to some extent. Another limitation of the study was the use of a non-validated health and lifestyle questionnaire. Self-reported data, such as socioeconomic status, physical activity and the nutritional knowledge level are not free of measurement errors. However, at the study design stage, we made an effort to ensure that the questions are comprehensibly presented to respondents by, for example, defining each physical activity level or using additional control questions on food expenditure. Moreover, we tested a limited number of determinants; unmeasured or residual confounding cannot be disregarded, and it is probable that some important factors of DS usage could have been omitted in our study. Another limitation was not a random but volunteered selection of respondents in the study. This resulted in an overrepresentation of women compared to men; women are generally more likely to participate in a nutritional type of research. Moreover, it is possible that students with a more health-oriented lifestyle were more likely to participate in the study as well.

## 5. Conclusions

Results of the study indicated that among university students, independent predictors of supplement use included being female, being physically active, having a normal body weight, the presence of chronic diseases, and non-smoking. In addition, nutritional knowledge and factors related to eating habits were positively associated with DS use, i.e., the usage of a special diet, eating more meals per day, consuming fortified food and following a Mediterranean-style diet. Identifying the factors affecting the use of DS provides an opportunity to adapt nutritional education to young people’s needs. Given that the use of vitamins and/or minerals is common in university students, and their users are characterized by eating a higher quality diet, investigating the contribution of DS in overall dietary nutrient intake warrants further study.

## Figures and Tables

**Table 1:** Socio-demographic and lifestyle parameters stratified by dietary supplement use in university students (n = 2545).

Parameters	DS Users ^a^	DS Non-Users	*p*-Value ^b^
	n = 1044	n = 1501	
Age (mean ± SD)	22.1 ± 2.5	22.2 ± 2.3	0.206
Gender (%)			
Female	82.2	73.9	<0.001
Male	17.8	26.1	
Living place (%)			
Village	17.7	21.5	0.153
Small town	19.7	19.3	
City	62.6	59.2	
Socioeconomic status (%)			
Very good or good	68.7	68.2	0.807
Average or poor	31.3	31.8	
Physical activity			
Low	35.9	43.5	<0.001
Moderate	34.8	37.7	
High	29.3	18.8	
Body mass index (kg/m^2^) (%)			
<18.5	9.9	8.9	0.139
18.5–24.9	75.0	72.0	
≥25	15.1	19.1	
Health status (%)			
At least good	85.8	88.5	0.042
Average or poor	14.2	11.5	
Cigarette smoking (%)			
No	88.4	81.5	<0.001
Yes	11.6	18.5	
Current chronic diseases (%)			
No	76.3	88.9	<0.001
Yes	23.7	11.1	
Special diet (%)			
No	73.3	86.9	<0.001
Yes	26.7	13.1	
Nutritional knowledge (%) ^c^			
Low	6.7	14.7	<0.001
Average	39.2	44.9	
High	51.8	38.5	
Number of meals/days (%) ^c^			
≤3	14.6	20.5	<0.001
4	38.6	30.8	
≥5	17.2	13.5	
Fortified food consumption (%) ^c^			
No	26.0	33.5	<0.001
Yes	54.7	45.0	

^a^ A DS user was considered a person who used at least one supplement for at least one week or longer over the past 6 months; ^b^ the Student’s t-test for continuous variables and the chi-squared test for categorical variables; ^c^ missing data/do not know: number of meals per day (32.9%), nutritional knowledge level (2.0%); fortified food consumption (20.6%); DS–dietary supplements; SD–standard deviation.

**Table 2:** The prevalence of specific types of dietary supplement use and reasons for using or non-using them by university students (n = 2545).

Parameters	DS Users ^a^ n = 1044	DS Non-Users n = 1501
	%	%
Type of DS ^b^		
Single vitamin	60.3	-
Single mineral	21.6	-
Multivitamin/mineral(s)	36.9	-
Vitamin(s)/mineral(s) + herbs	11.2	-
PUFA	16.7	-
Protein or protein-carbohydrate	15.0	-
Other	12.4	-
Usage more than one DS	45.7	-
Reason for using DS ^b^		
Improve overall health	65.2	-
Diet poor in nutrients	51.3	-
Medical recommendation	39.2	-
Improves memory and concentration	10.5	-
Necessary when medicines are used	3.2	-
Increase in strength and muscle mass	3.2	-
Beauty of hair, nail, and skin	2.8	-
Other	8.1	-
Reason for non-using DS ^b^		
No need to use because of proper nutrition	-	48.8
Lack effect on health improvement	-	10.7
High price	-	10.0
Can be harmful	-	7.9
Other	-	7.7

^a^ DS user was considered a person who used at least one supplement for at least one week or longer over the past 6 months; ^b^ more than one answer could be selected; missing data on the type of DS (0.7%); DS–dietary supplements; PUFA-polyunsaturated fatty acids.

**Table 3:** The logistic regression of dietary supplement use by socio-demographic and lifestyle determinants in university students (n = 2545).

Study Factors	Model 1 OR (95% CI)	Model 2 OR (95% CI)	Model 3 OR (95% CI)
Age (years)	0.98 (0.95–1.01)	1.00 (0.96–1.04)	
*p* for trend	0.21	0.97	
Gender			
Female	1.00	1.00	1.00
Male	0.62 (0.51–0.75)	0.63 (0.50–0.79)	0.62 (0.50–0.79)
Living place			
Village	1.00	1.00	
Small town	1.24 (0.90–1.70)	1.26 (0.90–1.77)	
City	1.29 (0.99–1.67)	1.18 (0.90–1.56)	
Socioeconomic status			
Very good or good	1.00	1.00	
Average or poor	0.98 (0.83–1.16)	1.13 (0.94–1.36)	
Physical activity			
Low	1.00	1.00	1.00
Moderate	1.11 (0.93–1.34)	1.08 (0.89–1.32)	ns
High	1.88 (1.53–2.31)	1.89 (1.49–2.40)	1.79 (1.45–2.20)
Body mass index (kg/m^2^)			
<18.5	1.07 (0.81–1.40)	1.14 (0.85–1.53)	ns
18.5–24.9	1.00	1.00	1.00
≥25	0.76 (0.61–0.94)	0.77 (0.60–0.97)	0.77 (0.61–0.98)
*p* for trend	<0.001	0.003	0.005
Health status			
At least good	1.00	1.00	
Average or poor	1.28 (1.01–1.62)	1.22 (0.92–1.61)	
Cigarette smoking			
No	1.00	1.00	1.00
Yes	0.58 (0.46–0.73)	0.67 (0.52–0.86)	0.67 (0.52–0.85)
Current chronic diseases			
No	1.00	1.00	1.00
Yes	2.48 (1.99–3.07)	2.30 (1.82–2.90)	2.37 (1.89–2.98)
Nutritional knowledge			
Low	1.0	1.0	1.00
Average	1.92 (1.43–2.57)	1.49 (1.08–2.05)	1.49 (1.09–2.04)
High	2.96 (2.20–3.96)	2.07 (1.49–2.88)	2.09 (1.51–2.88)
Special diet			
No	1.00	1.00	1.00
Yes	2.42 (1.97–2.96)	1.97 (1.58–2.45)	2.00 (1.61–2.49)
Number of meals/days			
≤3	1.00	1.00	1.00
4	1.76 (1.39–2.23)	1.49 (1.15–1.92)	1.32 (1.09–1.61)
≥5	1.80 (1.36–2.38)	1.28 (0.94–1.73)	ns
Fortified food consumption			
No	1.00	1.00	1.00
Yes	1.57 (1.30–1.89)	1.65 (1.35–2.01)	1.64 (1.35–2.00)

OR–odds ratio; CI–confidence interval; ns–not statistically significant in Model 3. Model 1–univariate model (crude data), Model 2–multivariate-adjusted model, Model 3–stepwise regression model.

**Table 4:** The mean daily energy, nutrients, and food consumption among university students by using dietary supplements (DS) (n = 1805).

Nutrient and Food Intake	DS Users n = 762	DS Non-Users n = 1043	*p*-Value ^a^
	Mean ± SD		
Energy (kcal)	1767 ± 505	1817 ± 568	0.109
Fat (% kcal)	31.4 ± 6.5	31.2 ± 6.4	0.772
Carbohydrates (% kcal)	47.1 ± 7.5	48.0 ± 6.8	0.021
Protein (% kcal)	18.0 ± 3.9	17.5 ± 3.3	0.004
Vegetables (g)	312 ± 199	256 ± 162	<0.001
Legumes, seeds, nuts (g)	25.2 ± 42.3	14.5 ± 24.4	<0.001
Fruits (g)	253 ± 177	232 ± 171	0.008
Cereals (g)	180 ± 71	191 ± 73	0.002
Fish and seafood (g)	19.8 ± 30.0	18.5 ± 29.7	0.310
Monosaturated to saturated fatty acids ratio	1.24 ± 0.43	1.19 ± 0.37	0.014
Dairy products (g)	232 ± 144	230 ± 132	0.887
Meat and meat products (g)	112 ± 106	119 ± 83.0	<0.001
Ethanol intake (g)	1.90 ± 0.32	2.15 ± 0.39	0.377
Mediterranean Diet Score (points)	5.16 ± 1.43	4.78 ± 1.42	<0.001

^a^ the Mann–Whitney U test; DS–dietary supplements; SD–standard deviation.

**Table 5:** The likelihood of using dietary supplements across quartiles of food consumption and the Mediterranean Diet Score (MDS) in university students (n = 1805).

Mediterranean-Style Diet Components	Quartile of Food Consumption Multivariate-Adjusted OR (95% CI) ^a, b^				*p*-Trend
	1	2	3	4	
Vegetables	1.00	0.89 (0.66–1.20)	1.08 (0.80–1.46)	1.76 (1.29–2.42)	0.002
Legumes, seeds, nuts	1.00	1.45 (1.07–1.96)	1.35 (1.01–1.82)	2.01 (1.47–2.76)	0.012
Fruits	1.00	0.99 (0.73–1.33)	0.91 (0.67–1.24)	0.67 (0.49–0.92)	0.005
Cereals	1.00	0.99 (0.74–1.32)	0.85 (0.63–1.14)	0.99 (0.73–1.33)	0.794
Fish and seafood	1.00 ^c^		0.88 (0.68–1.15)	0.91 (0.71–1.18)	0.328
Monosaturated to saturated fatty acids ratio	1.00	1.05 (0.90–1.21)	1.10 (0.99–1.22)	1.04 (0.96–1.12)	0.796
Dairy products	1.00	0.73 (0.54–0.99)	0.75 (0.55–1.01)	0.90 (0.67–1.23)	0.684
Meat and meat products	1.00	1.05 (0.78–1.42)	0.95 (0.70–1.29)	1.23 (0.90–1.67)	0.220
Ethanol intake	1.00 ^c^		1.19 (0.85–1.66)	1.10 (0.86–1.40)	0.393
	**Mediterranean-style diet adherance, MDS points**				
**DS usage**	**1–3** (n = 312)	**4** (n = 391)	**5** (n = 448)	**6–9** (n = 654)	
No (%) Yes (%)	67.3 32.7	60.9 39.1	59.6 40.4	50.2 49.8	<0.001 ^d^
Multivariate-adjusted OR (95% CI) ^a^	1.00	1.24 (0.89–1.73)	1.12 (0.81–1.55)	1.42 (1.04–1.93)	0.011

DS–dietary supplements, OR–odds ratio, CI–confidence interval. ^a^ Logistic regression models were adjusted for gender, physical activity, Body Mass Index, smoking status, current chronic diseases, nutritional knowledge, special diet, number of meals, fortified food consumption; ^b^ mutually adjusted for food groups that constitute the Mediterranean-style diet components; ^c^ a reference category includes the two lowest quartiles due to a lack of consumption of these products in the first and the second quartile; ^d^ *p*-value was calculated using the chi-squared test.

## Data Availability

The datasets generated for this study are available on request to the corresponding author.

## Supplementary Materials

The following supporting information can be downloaded at: https://www.mdpi.com/article/10.3390/nu14132745/s1, Health and Lifestyle Questionnaire; Table S1: A sex-specific median intake cut-off of Mediterranean diet components and scoring system (n = 1805).
