# Supplementary material for: Dietary Supplement Use in Relation to Socio-Demographic and Lifestyle Factors, including Adherence to Mediterranean-Style Diet in University Students
Source: Nutrients. 2022 Jun 30;14(13):2745. doi: 10.3390/nu14132745 (PMC9269239; doi:10.3390/nu14132745)
Supplement: Supplementary file 1 [file nutrients-14-02745-s001.zip › nutrients-1772615-supplementary.pdf]

WARSAW UNIVERSITY OF LIFE SCIENCES
Department of Human Nutrition
159 C Nowoursynowska St., 02-776 Warsaw

Date......................   **Respondent no.** ....

HEALTH AND LIFESTYLE QUESTIONNAIRE

I kindly ask you to participate in a study to assess the use of dietary supplements and the consumption of products with added vitamins and/or minerals among students.

Section I. Socio-demographic characteristics

1. Gender
□ **Male**
□ **Female,** are you currently pregnant or breastfeeding?   □ no
□ yes, in (which?) ___week of pregnancy   □ yes, I am breastfeeding

2. Age ....

3. Field of study...

4. Place of residence:
□ city
□ small town
□ village

5.1. Do you live independently?
□ no (in the family home)   □ yes (e.g. student hostel, student residence, own apartment)

5.2. How many people live in your household, including yourself?
Total number of people: ....

6.1. How would you rate your financial situation? *(please mark one answer)*
□ very good   □ good   □ average
□ bad   □ difficult for me to judge

6.2. Please give an approximation of your weekly food expenses:
□ food consumed at home: approx. ______ PLN
□ food consumed outside of home: approx. ______ PLN
□ I don't know

Section II. Health and lifestyle status

7. How do you evaluate your overall health? *(please mark one correct answer)*
□ excellent
□ very good
□ good
□ not bad
□ bad

8. Have you been diagnosed with any chronic diseases?
□ no □ yes
If yes, what type of chronic disease do you suffer from?...
....

9. Have you used any medications regularly during the past month?
□ no □ yes

If "yes", please complete the table below regarding the medications you are taking (for women: please include oral contraceptives).

Name of drug	For what disease	Single dose	Number of doses per day

10. Do you smoke cigarettes, cigars, or tobacco in any other form?
□ no, I have never smoked
□ no, I do not currently smoke, but I have smoked in the past
□ yes, I am currently smoking

11. Please specify your physical activity including sports, type of occupation, leisure activities, etc.:
□ **very low** (sedentary lifestyle, sometimes short walks or other activity)
□ **low** (light exercise such as walking or other activity) **-** at least 2-4 hours per week
□ **medium** (exercise such as running, walking uphill, swimming, fitness, gym, playing ball, cycling) for 1-2 hours per week or low intensity more than 4 hours per week
□ **high** (intense exercise as above) more than 3 hours per week
□ **very high** (intense exercise regularly) several times a week

12.1. Current weight: ____ kg **12.2. Height: ___ cm**

Section III. Nutritional knowledge and eating habits

13. How would you rate your nutritional knowledge? *Please mark one answer that best represents your opinion:*
□ no knowledge at all
□ no knowledge
□ rather no knowledge
□ neither lack of knowledge nor having knowledge
□ rather having knowledge
□ good knowledge
□ excellent knowledge

14. Are you currently following or have you followed any diet in the past month?
□ no
□ yes
□ medical (prescribed by a doctor)
□ weight-loss diet
□ vegetarian
□ other, what kind?..

How long have you been following this diet? .... months/...... years

15. Do you think that you eat properly on a daily basis?

□ no □ yes □ it is difficult to judge

16. How many meals a day do you usually eat? *(please mark one appropriate answer)*

□ 1-2 □ 3 □ 4 □ 5 and more

17. Do you eat meals regularly? *(please answer by putting an "x" in each row)*

	No	Yes	Irregular
Breakfast			
Morning snack			
Lunch			
Afternoon snack			
Dinner			

18. Do you snack between meals:

□ no
□ yes, regularly
□ sometimes

19. Are there foods that you deliberately exclude from your diet?

□ no
□ yes, which foods does this apply to?...
....

20. Are there foods that you intentionally include in your diet?

□ no
□ yes, which foods?...
....

21. Do you drink alcohol?

□ no, never
□ yes, occasionally: once a week or less
□ yes, daily or almost daily
□ yes, twice to three times a week

Section IV. Dietary supplements usage

22. Do you use any dietary supplements currently or have you used them in the past 6 months?
(e.g. vitamin-mineral products, vitamin D, magnesium, cod liver oil, etc.)

□ no *(if no, go to **question no. 25**)* □ yes

If "yes", please fill in the table below regarding the products you are taking.

**Supplement name**	**Brand**	**Form** (capsules, tablets, powder, etc.)	**Duration of use** (how many days, weeks, months)	**Single-dose** (how many tablets, drops, etc.)	**Number of doses used per week**	**By whose recommendation** (own decision, doctor, etc.)

23.1. If „yes", when do you usually take dietary supplements?
□ on an empty stomach
□ after a meal
□ during a meal
□ variously

23.2. If you have consumed more than one supplement, do you use or have used these supplements during the same period (e.g. on the same day, week, etc.)?
□ no
□ yes, which products? ....
....

23.3. Are you currently using these supplements?
□ no
□ yes, which ones? ....
....

24. What is your reason for using dietary supplements? (*you can choose more than one answer*)
□ diet poor in nutrients
□ to improve overall health
□ to improve memory and concentration
□ necessary when using medications
□ medical recommendation
□ other, what? ....

please go to ***question 26***

Question 25 is only for those who answered "no" to question 22
25. You stated that you have not used dietary supplements in the last 6 months. What is your reason for non-using dietary supplements? (*you can choose more than one answer*)
□ lack of effect on health
□ no need to use because of proper nutrition
□ can be harmful to your health
□ are too expensive
□ other, what? ....

Section V. Fortified foods usage

26. Do you include fortified foods in your habitual diet?
Products which on the label have written that they contain added nutrients; the ingredients have been voluntarily added by the producers at the product manufacturing stage to enrich their nutritional values, e.g. cereal with vitamins, yogurt with calcium and vitamin D.

□ no, *please go to* ***question 29***
□ yes, *please answer* ***questions 27-28***
□ I don't know

27. What is your reason for consuming fortified foods? *(you can mark more than one answer)*
□ beneficial effect on health
□ taste preferences
□ doctor/pharmacist recommendation
□ diet contains too few of the ingredients in which the product is enriched
□ other, what? ....

28. What is your source of information about voluntarily fortified foods?
....
....

Question 29 is only for those who answered "no" to question 26
29. What is your reason for avoiding fortified foods? *(you can mark more than one answer)*
□ lack of impact on health
□ eating properly and no need for such products
□ too expensive
□ bad taste
□ other, what? ....
....

30. When choosing a product, do you pay attention to it being fortified?
□ no □ yes □ I don't know

31. Does the fact that a product is fortified affect your decision to buy it?
□ it has no effect
□ yes, I choose such a product
□ yes, I am not buying such products
□ I don't know

Thank you for completing the survey!

Supplementary Material Table S1. A sex-specific median intake cut-off of Mediterranean diet components and scoring system (n = 1 805)

Mediterranean Diet Components [a]	Median intake (P5-P95)		Dietary scoring (1 point assigned)
	Women (n=1399)	Men (n=406)	
Vegetables (g)	238 (76 – 592)	257 (88 – 681)	≥ median
Legumes, seeds, nuts (g)	8.0 (0 – 76)	2.8 (0 – 62)	≥ median
Fruits (g)	230 (20 – 565)	155 (0 – 541)	≥ median
Cereals (g)	168 (83 – 270)	230 (106 – 405)	≥ median
Fish and seafood (g)	0 (0 – 74)	0 (0 – 83)	≥ median
Monosaturated to saturated fatty acids ratio	1.13 (0.70 – 1.98)	1.16 (0.78 – 1.90)	≥ median
Dairy products (g)	213 (38.5 – 464)	214 (41 – 486)	< median
Meat and meat products (g)	84 (0 – 204)	179 (54 – 401)	< median
Etanol intake (g)	0 (0 – 9)	0 (0 – 18)	Women: ≥5 and ≤25 g/day<br>Men: ≥10 and ≤50 g/day
MDS (points)	5.0 (3.0 – 7.0)	4.0 (2.0 – 7.0)	

[a] The Mediterranean diet score (MDS) can take values from 0 (a lack of adherence) to 9 points (a high adherence to the Mediterranean-style diet) [18]; Abbreviations: P5-P95, 5th and 95th percentile of intake.
